# Predictors for Developing Severe Myalgic Encephalomyelitis/Chronic Fatigue Syndrome Following Infectious Mononucleosis

**DOI:** 10.29245/2767-5122/2021/1.1129

**Published:** 2022-02-21

**Authors:** Leonard A Jason, Joseph Cotler, Mohammed F Islam, Jacob Furst, Ben Z Katz

**Affiliations:** 1 Center for Community Research, Depaul University, Chicago, IL 60614, USA.; 2 Department of Psychology, Chicago State University; 3 The College of Computing and Digital Media, Depaul University, Chicago, IL 60614, USA.; 4 Northwestern University Feinberg School of Medicine, Department of Pediatrics, Chicago, 60611 USA.

**Keywords:** Cytokines, Infectious Mononucleosis, Myalgic Encephalomyelitis / Chronic Fatigue Syndrome, Chronic Fatigue Syndrome, Myalgic Encephalomyelitis

## Abstract

**Background:**

About 10% of individuals who contract infectious mononucleosis (IM) have symptoms 6 months later that meet criteria for myalgic encephalomyelitis/chronic fatigue syndrome (ME/CFS). Our study for the first time examined whether it is possible to predict who will develop ME/CFS following IM.

**Methods:**

We have reported on a prospectively recruited cohort of 4,501 college students, of which 238 (5.3%) developed IM. Those who developed IM were followed-up at six months to determine whether they recovered or met criteria for ME/CFS. The present study focuses on 48 students who after six months had a diagnosis of ME/CFS, and a matched control group of 58 students who had no further symptoms after their IM. All of these 106 students had data at baseline (at least 6 weeks prior to the development of IM), when experiencing IM, and 6 months following IM. Of those who did not recover from IM, there were two groups: 30 were classified as ME/CFS and 18 were classified as severe ME/CFS. We measured the results of 7 questionnaires, physical examination findings, the severity of mononucleosis and cytokine analyses at baseline (pre-illness) and at the time of IM. We examined predictors (e.g., pre-illness variables as well as variables at onset of IM) of those who developed ME/CFS and severe ME/CFS following IM.

**Results:**

From analyses using receiver operating characteristic statistics, the students who had had severe gastrointestinal symptoms of stomach pain, bloating, and an irritable bowel at baseline and who also had abnormally low levels of the immune markers IL-13 and/or IL-5 at baseline, as well as severe gastrointestinal symptoms when then contracted IM, were found to have a nearly 80% chance of having severe ME/CFS persisting six months following IM.

**Conclusions:**

Our findings are consistent with emerging literature that gastrointestinal distress and autonomic symptoms, along with several immune markers, may be implicated in the development of severe ME/CFS.

Myalgic encephalomyelitis/chronic fatigue syndrome (ME/CFS) is a debilitating illness affecting over a million people in the US^1^. Six months after having had Infectious Mononucleosis (IM), about 9–12% of individuals are diagnosed with this syndrome. For example, Hickie et al.^2^ showed an 11% rate of ME/CFS six months following acute infection with Epstein-Barr virus as well as following two other similar systemic infections. Katz et al.^3^ found similar outcomes following IM in youth.

In a recent prospective, longitudinal study, university students were assessed prior to IM, at the time of IM, and at a six-month follow-up. Those who developed severe ME/CFS had differences in several pre-illness domains compared to those who recovered from IM without further symptoms^4^. In addition, Katz et al.^5^ found that the severity of IM was predictive of severe ME/CFS 6 months following IM. We have used these baseline pre-illness behavioral and immune data along with severity of IM data to predict who will develop severe ME/CFS six months following IM.

## Methods

### Participants, Setting, and Procedures

Jason *et al.* (2021)^4^ provide demographic characteristics and details of the 4,501 Northwestern University students who were enrolled in our previously reported prospective, longitudinal study. The students were consented, completed seven questionnaires including the DePaul Symptom Questionnaire (DSQ, a self-report measure of ME/CFS symptomatology^6^) and the MOS 36-item Short-Form Health Survey^7^ and had blood samples taken. Students’ IM was diagnosed and tracked by the Northwestern University Health Service and other medical providers. IM was defined as a positive monospot or specific Epstein-Barr virus serologies (a positive viral capsid antigen [VCA] IgM or a positive VCA IgG with a negative EB nuclear antigen antibody) in the appropriate clinical setting. Those who developed IM at least six weeks after enrollment again provided online consent, completed the same questionnaires as during the baseline phase, and had blood samples taken. Five months after the IM diagnosis, students deemed not recovered and an approximately equal number deemed recovered via a phone screen were invited to participate in a six-month assessment; these participants completed the same measures again, and in addition underwent a comprehensive medical examination. Institutional Review Board approval was obtained. Our present study focused on 48 students who, 6 months following IM, still had symptoms of ME/CFS, and a matched control group of 58 who had no further symptoms after their IM. Therefore, our current study involved 106 students, and these students had data at baseline, data from when they had IM, and data 6 months following IM.

### ME/CFS Diagnosis

Participants’ six-month medical examination, and the results from their DePaul Symptom Questionnaire^6^ and MOS 36-item Short-Form Health Survey^7^ were used in the diagnosis of ME/CFS. While there are multiple ME/CFS case definitions^8–11^, we selected three major ones including the Fukuda et al. criteria^8^, the Canadian Consensus criteria^9^ and the Institute of Medicine criteria^11^. A number of studies^12–14^ have found that the Fukuda et al. criteria are broader and identify a larger group of patients, with less severity, than those identified by the Canadian Consensus or Institute of Medicine criteria. Therefore, participants who primarily only met the Fukuda et al.^8^ criteria were defined as having ME/CFS. Those who met more than one case definition (i.e., the Fukuda and either the Canadian and/or Institute of Medicine criteria) were defined as having *severe* ME/CFS. Those who met more than one case definition also scored worse on the DSQ than those who met only a single case definition for ME/CFS six months following IM. Those who recovered were labeled controls. For participants with data at all stages of the study, 30 were classified as ME/CFS, 18 were classified as severe ME/CFS, and 58 were classified as controls.

### Severity of Mononucleosis

Katz et al.^5^ used a validated severity of mononucleosis scale to categorize the students who developed IM. The scale was based on the literature and each severity item was assigned a score of 1. The gastrointestinal (GI) symptoms that were part of this questionnaire included anorexia, nausea and vomiting; these symptoms were the main drivers of mononucleosis severity and were thus examined in more detail in the current study.

### Cytokines

For cytokine analysis, stored plasma was used. The following cytokines were evaluated using a multiple analyte platform and commercially customized kits from Millipore (Billerica, MA): IL1α, IL1β, IL2, IL4, IL5, IL6, IL8, IL10, IL12(p70), IL13, IL15, IL17α, IL23, IFN-γ, TNF-α and TNF-β. Each plasma sample was run in duplicate.

### Statistical analysis

Receiver Operating Characteristic (ROC) statistics were calculated using combination predictor variables to designate severe ME/CFS outcomes. When we tried to use the statistical methods to predict the development of ME/CFS following IM, the results were not significant, so in our analyses reported below, we focused on the severe ME/CFS group. Our first ROC analysis took the significant variables from prior studies, including the Total DSQ, three immune markers (IL-5, IL-6, and IL-13) found to differ between controls and those with severe ME/CFS at baseline^4^ and the score on the severity of mononucleosis scale (0, 1 or > 1, the latter of which was predictive of the development of severe ME/CFS 6 months later^5^).

Our second ROC analysis first identified specific features by using random forest analyses^15^ from the random forest R packages (https://cran.r-project.org/web/packages/randomForest/randomForest.pdf). The 54 items from the DePaul Symptom Questionnaire and the 16 cytokines were standardized and centered. Two random forest classification analyses were conducted to separate patients with severe ME/CFS from controls: one with just the clinical DSQ items, and one with just the cytokines. The DSQ items with the largest decrease in gini impurity (a metric assessing the accuracy of a single feature) were summed to create a clinical metric, while the cytokines with the largest decrease in gini impurity were combined to create a metric for the cytokines. Stomach pain, bloating, and irritable bowel were the three-baseline clinical DSQ features found to be the most predictive, while decreased levels of IL-5 and IL-13 were the two baseline immune cytokines found to be the most predictive^4^. Therefore, these baseline features were included as predictive features. As the selected features were all gastrointestinal in nature, as were the severe symptoms at the time of mononucleosis from the severity of mononucleosis scale^5^, the latter were also selected as a predictor. An index was then created using the selected features, yielding a 0–24 scale for the three baseline DSQ items as follows: the frequency and severity of bloating (both rated on a scale of 0–4), the frequency and severity of stomach pain (both rated on a scale of 0–4), and the frequency and severity of symptoms of an irritable bowel (e.g., gas or changes in stool [i.e., diarrhea or constipation], both rated on a scale of 0–4). The gastrointestinal DSQ symptoms were standardized, centered to 0, and summed to create the clinical metric. Whether the participant also experienced severe gastrointestinal symptoms when they had mononucleosis was rated as a binary outcome (1=absent, 2=present); the clinical metric score was then multiplied by this outcome, so that if the participant experienced severe gastrointestinal symptoms at the time of mononucleosis, the clinical metric was doubled. Finally, the immune metric was subtracted from the clinical metric due to the inverse relationship between these anti-inflammatory cytokines and severe ME/CFS^4,16^, to arrive at the final index.

## Results

**Figure 1.**
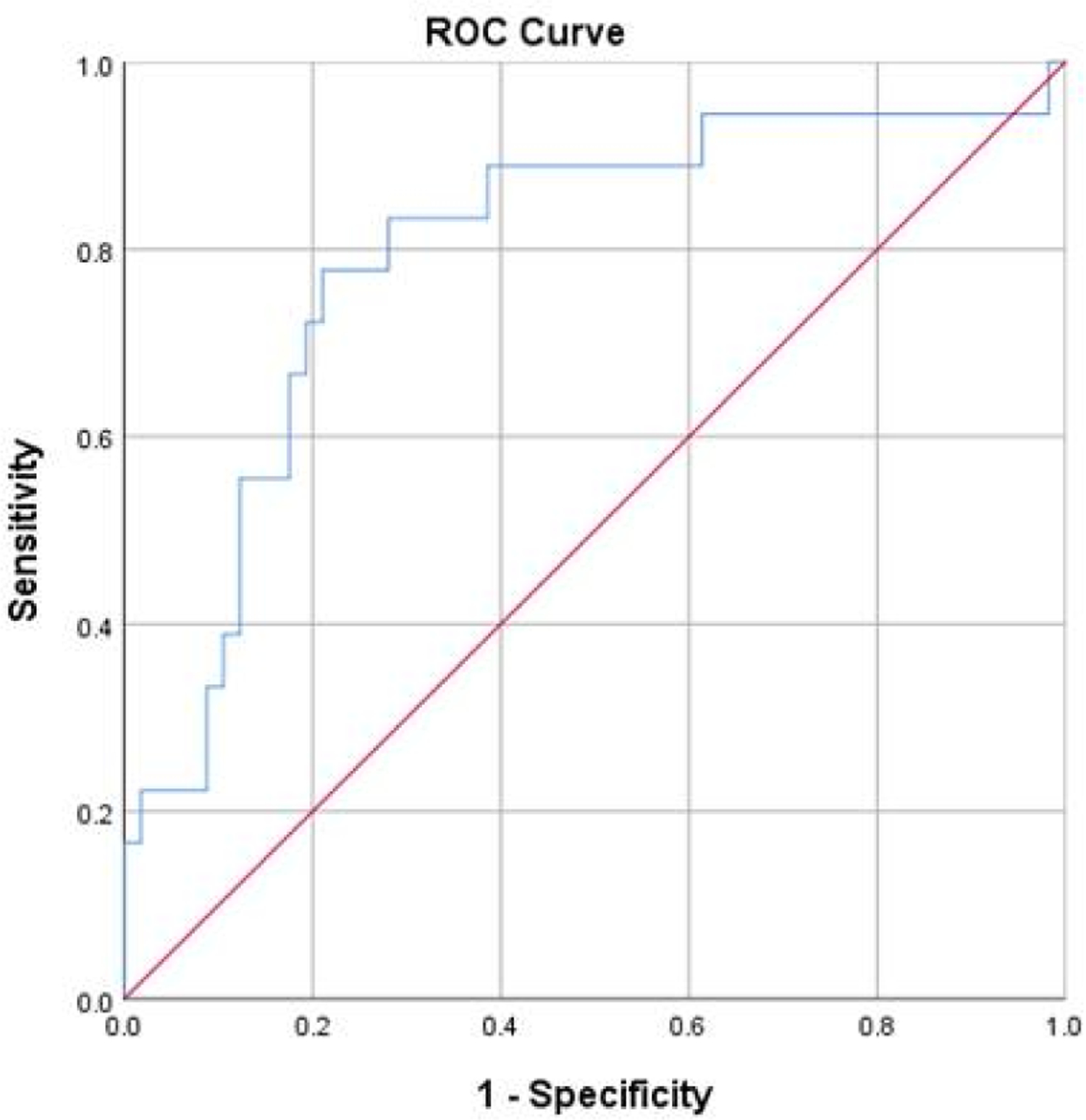
ROC Curve Predicting Severe ME/CFS from 6 Marker Variables^1,2^ ^1^Comparing 18 students classified with severe ME/CFS versus 58 classified as controls. ^2^Predictor variables: At baseline, low levels of IL-13 and/or IL-5 and stomach pain, bloating, and symptoms of an irritable bowel; at the time of IM, severe gastrointestinal symptoms.

The first ROC analysis data, which used baseline DSQ total data, the three immune markers and the Severity of Mononucleosis Scores at the time of IM, yielded an AUC = .676 (*p* =.025; *SE* = .084, 95% CI [.511, .842]). A score of −0.25 would have a sensitivity of 0.722 and a 1-specificity of 0.47, which are inadequate for prediction purposes. We therefore next examined specific features of our second ROC analysis, which is displayed in Figure 1. The index included six variables: the three baseline DSQ items that differentiated the groups (stomach pain, bloating, and symptoms of an irritable bowel), the two baseline cytokines (IL-13 and/or IL-5) that were decreased, and whether or not the patient experienced severe gastrointestinal symptoms at the time they contracted mononucleosis. The AUC for this second ROC analysis was .795 (*p* < .01; *SE* = .064, 95% CI [.669, .921]). A score of 2.08 had a sensitivity of .778 and a 1 - specificity of .211 for predicting severe ME/CFS. This indicates that patients with stomach pain, bloating, and symptoms of an irritable bowel at baseline, severe gastrointestinal symptoms when they contracted mononucleosis, and low levels of IL-13 and/or IL-5 at baseline had nearly an 80% chance of developing severe ME/CFS six months following IM.

## Discussion

The current study indicated that it is possible to use pre-illness and illness variables to predict the occurrence of severe ME/CFS following IM. We showed that those who develop severe ME/CFS 6 months following IM had more baseline gastrointestinal symptoms and abnormal immune markers and had worse gastrointestinal symptoms at the time they developed IM. These signs and symptoms at baseline and at the time of IM thus appear to predict the development of severe ME/CFS 6 months following IM and allowed us to develop a model that, if validated in a different population, might be generally useful in predicting the development of severe ME/CFS.

Our study also shows the importance of gastrointestinal symptoms for the development of severe ME/CFS. It is of interest that the original Fukuda et al.^8^ case definition did not include gastrointestinal or autonomic symptoms among those that qualify for a diagnosis of ME/CFS. Gastrointestinal symptoms such as nausea, bloating and vomiting may be linked to autonomic dysfunction. Our findings are also thus consistent with emerging literature that gastrointestinal distress and autonomic symptoms should be considered important symptom categories for ME/CFS^17–19^; similarly, Jason et al.^20^ and Katz et al.^21^ found that days spent in bed since infectious mononucleosis, along with autonomic dysfunction two months after the diagnosis, were associated with postinfectious ME/CFS at six months. In addition, IL-13 and IL-5 were implicated as risk factors for the development of severe ME/CFS following IM, and there is evidence in human and mouse models that IL-5 and IL-13 contribute to the pathology associated with ulcerative colitis^22,23^; IL-13 is thought to play a key role in the pathophysiology of ulcerative colitis and fistulizing Crohn’s disease, and IL-13 blockade is a promising therapeutic strategy for these diseases^24^ However, it should be noted that in patients with Crohn’s disease, there is an overproduction of IL-13 whereas in the subjects with severe ME/CFS in the present study the IL-13 is lowered.

Our study has a number of limitations, including a relatively small sample size for the severe ME/CFS group. In addition, the sample consists of college students, so it is unknown if the findings will generalize to other adult samples. Third, the model was only predictive of severe ME/CFS. Finally, it is not known whether the gastrointestinal distress during IM contributes to the subsequent symptoms of ME/CFS, or whether the gastrointestinal distress itself is caused by some other factor. The gastrointestinal distress at baseline and during IM may be indicative of dysbiosis of the gut microbiota, which has been shown to contribute to other chronic inflammatory conditions^25–27^.

It would be instructive to determine if these same predictors identified those predisposed to severe COVID-19 and/or “long-haul” COVID as well. Youth infected with SARS-CoV-2 do report more symptoms than controls at follow up^28^, and in a recent study dealing with recovery from COVID, reactivation of latent EBV and SARS-CoV-2 RNAemia were predictors of post-acute sequelae of covid-19 (PASC), and those reporting gastrointestinal PASC, SARS-CoV-2-specific and CMV-specific CD8+ T cells exhibited unique dynamics^29^.

We had previously found that those who developed ME/CFS following IM did not have any significant baseline differences in stress, coping, anxiety, or depression, but before IM they had several cytokine markers that were significantly different from those who did not develop ME/CFS following IM^4^. We concluded that deficiencies in the production of these cytokines prior to contracting IM may influence immune response and cause immune dysregulation once the virus is contracted. The current study for the first time attempted to predict onset of ME/CFS using pre-illness and illness data. If validated, these predictors of risk could significantly alter the therapeutic strategies in IM and other triggers of ME/CFS in adults and adolescents. In addition, our work may lead to a reappraisal of the ME/CFS diagnostic criteria to include objective gastrointestinal and/or autonomic criteria for diagnosis.
